# Epidemiological trends, predictive factors, and projection of tooth loss in Germany 1997–2030: part II. Edentulism in seniors

**DOI:** 10.1007/s00784-020-03265-w

**Published:** 2020-04-03

**Authors:** Falk Schwendicke, Ina Nitschke, Helmut Stark, Wolfgang Micheelis, Rainer A. Jordan

**Affiliations:** 1 Department of Operative and Preventive Dentistry, Charité – Universitätsmedizin Berlin, Aßmannshauser Str. 4-6, 14197 Berlin, Germany; 2 Clinic for General, Special Care and Geriatric Dentistry, Center of Dental Medicine, University of Zurich, Zurich, Switzerland; 3 Department of Prosthodontics, Preclinical Education and Dental Materials Science, Rheinische Friedrich Wilhelms University of Bonn, Bonn, Germany; 4 Institute of German Dentists (IDZ), Cologne, Germany

**Keywords:** Cross-sectional study, Demography, Epidemiology, Prediction model, Tooth loss

## Abstract

**Objective:**

This is the second part of a report on tooth loss in Germany 1997–2030. Here, we describe trends in the prevalence of edentulism in seniors 1997–2014, assess predictive factors for edentulism, and projected it into 2030.

**Material and methods:**

We used data from three waves of the cross-sectional, multi-center, nationwide representative German Oral Health Studies. Overall, 3449 seniors (65–74 years) were included (1997: 1367; 2005: 1040; 2016: 1042). Age, sex, educational level, smoking status, and the cohort were entered into age-cohort binary-logistic regression models to assess the association of predictors with edentulism and to project edentulism in 2030 via Monte Carlo simulations.

**Results:**

Between 1997 and 2014, the prevalence of edentulism decreased from 24.8 to 12.4%. With each year of age, the risk of being edentate increased (by 11%, *p* < 0.001); it was also significantly increased in female versus male (by 40%, *p* = 0.001), low versus medium and high educational level (up to 257%, *p* < 0.001), and in former and current smokers (up to 258%, *p* < 0.001). We predict the prevalence of edentulism to be reduced to 4.2% in 2030. The reduction will be higher in males, never and former smokers, and those with low socio-educational level. On an absolute level and despite a growing elderly population (aged 60–80 years), the number of edentate individuals will have decreased by 3.6 million in 2030 compared with 1997.

**Conclusions:**

Edentulism in seniors has declined equitably in Germany. The decline is expected to continue until 2030. Further efforts are needed to tackle the underlying risk factors.

**Clinical relevance:**

This study presents trends of edentulism in Germany for a period of three decades. It provides clinically relevant data for health care planning by 2030.

## Introduction

Tooth loss is the final outcome of dental diseases like caries and periodontitis, developing through complex pathways over an individual’s life course [1]. Tooth loss exceeding a certain number of teeth significantly affects masticatory function and nutrition, speech, and esthetics. This is the more true for complete tooth loss, that is edentulism, which significantly decreases the oral health related quality of life, even when managed using complete dentures [2–4]. Edentulism is an established indicator for a population’s oral health, as it is highly relevant for patients, and swiftly and reliably to record.

Between 1990 and 2010, the global prevalence of edentulism decreased from 4.4 to 2.4% after age standardization [5], following longer-term trends observed in many industrialized countries like the USA, where edentulism prevalence declined from 19% in the 1950s to 5% in the late 2000s. Notable, this decline was not equally distributed between populations, with high-income households experiencing a greater relative decline than low-income households (the absolute decline, however, was smaller). For the USA, a projection until 2050 found this decline to continue, albeit slower, but to be partially offset in absolute terms by population growth and aging [6].

For Germany, the longitudinal trends of edentulism have not been assessed so far. Understanding such trends and evaluating the distribution of edentulism in different populations and regions as well as projecting prevalence into the future are relevant from a public health perspective and for targeted healthcare policy. The present study is the second part of a report on tooth loss in Germany 1997–2030 [7]. Here, we aimed to describe trends in the prevalence of edentulism in seniors 1997–2014, to assess predictive factors for edentulism, and to project edentulism into 2030.

## Material and methods

This study follows the methodology described in part I of this report [7]. Again, we followed the TRIPOD statement [8].

### Source of data

The data of this report stemmed from three waves of the German Oral Health Studies (Deutsche Mundgesundheitsstudie, DMS); DMS III from 1997, DMS IV from 2005, and DMS V from 2014 [9–11]. Details on the DMS, which are cross-sectional, multi-center, and nationwide representative oral health studies, have been described in the first part of this report [7]. Here, we only report on the age group of 65–74 years old (“younger seniors”), as only here significant percentages of edentulism were reported (in the 12-year-old group, prevalence was 0%, while in the group of those aged 35–45 years, prevalence ranged between 0.8% and 1.0% over the three waves). Overall, 3449 younger seniors were included and examined in this study (DMS III: 1367; DMS IV: 1040; and DMS V: 1042).

### Predictor variables

For this study, our primary outcome was edentulism that is, having no teeth at all. The predictor variables for this outcome were recorded at inclusion of the patient using self-administered validated questionnaires. Note that for this study, we only used those predictors that were concurrently available in sociodemographic projections for 2030. The following variables were used: (1) age in years; (2) sex; (3) educational level as low, middle, or high; (4) smoking status as never, former, and current; and (5) the cohort (coded via study wave as dummy variable; DMS III, IV, and V). Data on the collection of these as well as missing data and the handling of it are described in the first part of this report [7].

### Statistical analysis and projection

As in the first part of the report, age-cohort regression models [12] were used for analysis and projection. Here, a binary logistic regression model was used; all predictors were entered jointly (only multivariable models were used). We also tested for a number of interaction terms, without the model having better fit. Exponentiated regression coefficients, that is odds ratios and 95% CI, were used to present the risk of edentulism. Details on validation and the software used can be found in the first part of this report [7].

### Projection 2030

To make projections of edentulism in 2030, we used the yielded regression coefficients from the validated model and applied them to predicted population of 60–80 years old in 2030. This population age range was chosen, as we expected edentulism to occur in relevant numbers from age 60 onwards, and as more recent data indicated a higher prevalence above age 80 years, which we could not account for given that this age group had only been assessed in the last wave of the DMS [13, 14]. To do so, predictor data were collected from a number of sources:

1. Predicted demographic data were yielded from the national statistical office [15].
2. Sex proportions in this age group were estimated at 54% female and 46% male in 2030 [15].
3. Educational level was assumed to be carried forward from the population aged 35–44 years in 1997 (as we did not assume educational level to change greatly after that age), with 30%, 41%, and 29% of individuals being in the low, medium, and high social group, respectively.
4. Smoking status was derived from predictions made by the WHO, with stratification for sex [16]. We assumed 20% (95% CI 17; 23) and 16% (95% CI 13; 19) of male individuals in this age group to be current and former smokers, respectively. The number for females was 15% (95% CI 12; 18) for both current and former smokers, respectively.
5. The cohort effect was introduced by extrapolating the dummy variables of each DMS wave.
6. Spreadsheet-based Monte Carlo simulations were used to project edentulism as described elsewhere [7].

Population level estimates were calculated using past and predicted demographic data from the national bureau of statistics [15], with absolute and relative differences over the 33 years-period 1997–2030 being calculated. The population level estimates were further sub-grouped into different sexes and educational levels as well as according to smoking status.

## Results

The overall characteristics of the sampled cohorts in 1997, 2005, and 2014 are displayed in Table 1. Both the proportion of individuals with low education and of current smokers decreased with time, while sex proportions remained relatively stable. The prevalence of edentulism decreased significantly from 24.9 to 12.4%. When evaluating edentulism in different dental arches, it became obvious that edentulism was a more frequent phenomenon in the upper than the lower arch regardless of the period of assessment, and that the relative reduction was similar in the upper arch (1997/2005/2014; mean (95% CI): 45.4 (42.8–48.1)/32.1 (29.2–34.9)/18.8 (16.4–21.2)%; mean reduction 1997–2014: − 59%) and the lower arch (29.2 (26.8–31.6)/23.8 (21.2–26.4)/12.3 (10.3–14.3)%; − 58%).

**Table 1:** Characteristics of included patients in different waves of the German Oral Health Study (DMS). Due to missing data, not all subgroups sum up to total *N* values

		DMS III (1997, *N* = 1367)		DMS IV (2005, *N* = 1040)		DMS V (2014, *N* = 1042)	
Parameter		*N*	%	*N*	%	*N*	%
Age in years (SD), min-max		69 (2)	65–74	69 (3)	65–74	69 (2)	65–74
Sex	Male	578	42.2%	480	46.2%	489	46.9%
	Female	789	57.8%	560	53.8%	553	53.1%
Educational level	Low	1031	75.9%	660	65.8%	642	63.9%
	Medium	183	13.5%	183	18.1%	190	18.9%
	High	145	10.7%	163	16.1%	173	17.2%
Smoking pattern	Never	815	60.1%	695	61.5%	545	52.5%
	Former	358	26.4%	305	29.9%	362	34.9%
	Current	182	13.5%	88	8.6%	131	12.6%
Edentulous	No	1027	75.1%	810	77.9%	913	87.6%
	Yes	340	24.8%	230	22.1%	129	12.4%

When entering the predictors into the model, all parameters were significantly associated with edentulism prevalence (Table 2). While there were no significant differences in prevalence (when adjusting for all other predictors) in 2005 compared with 1997, the risk of being edentate was significantly decreased (by more than 50%) in 2014 compared with 1997. With each year of age, the risk increased (in mean by 11%); it was also significantly increased in female versus male (by 40%), low versus medium and high educational level, and in former and, more so, current smokers (for the latter, it was more than tripled). The model was robust when split-sample coefficients were tested in the other half of the sample (the predicted prevalence in the different waves deviated by < 0.5% when compared with reported prevalence).

**Table 2:** Multivariable analysis (*N* = 699 edentulous per 3.448 total; log-likelihood: 3068, *R*^2^ = 0.13)

Parameter	Category	OR	95% CI	*p* value
Cohort (ref: 1997)	2005	0.93	0.76–1.14	0.487
	2014	0.42	0.33–0.54	< 0.001
Age	Per year	1.11	1.08–1.15	< 0.001
Gender (ref: male)	Female	1.40	1.15–1.70	0.001
Educational level (ref: low)	Medium	0.39	0.29–0.52	< 0.001
	High	0.28	0.19–0.40	< 0.001
Smoking (ref: never)	Former	1.48	1.18–1.86	0.001
	Current	3.58	2.76–4.65	< 0.001

Using the coefficients shown in Table 2, the mean predicted prevalence of edentulism in those aged 60–80 years for 2030 was 4.2% (95% CI 2.8; 5.8), which was less than half of that in 2014 (Table 3). When assessing population level estimates, it was obvious that despite a growing population aged 60–80 years in Germany, those without own teeth will have decreased by 80% (or, in absolute terms, 3.6 million) compared with 1997, and > 65% compared with 2014. The reduction was higher in male than in female, never and former than current smokers, and those with low than medium and high educational level (Table 3).

**Table 3:** Population level estimates of edentulism in 1997 until 2030, and relative and absolute changes between 2030 and 1997. Note that only means are reported. Total N: total population aged 60–80 years, edent. N: total edentulous population, edent. %: prevalence of edentulism. Due to rounding, values in subgroups do not necessarily add up to total values

Parameter		1997			2005			2014			2030			Prevalence decline	
		Total (million)	Edent. (million)	Edent. (%)	Total (million)	Edent. (million)	Edent. (%)	Total (million)	Edent. (million)	Edent. (%)	Total (million)	Edent. (million)	Edent. (%)	Absolute (million)	Relative (%)
Total		18.2	4.5	24.9	20.5	4.5	22.1	21.8	2.7	12.4	21.9	0.9	4.2	3.6	79.7
Sex	Male	9.0	2.0	22.4	10.1	2.0	19.6	10.6	1.3	11.9	9.6	0.4	3.9	1.6	81.4
	Female	9.2	2.5	26.7	10.4	2.6	24.6	11.2	1.4	12.8	11.3	0.5	4.5	1.9	79.3
Educational level*	Low	13.7	3.8	27.7	13.5	3.7	27.3	10.5	1.7	16.4	6.6	0.5	8.1	3.2	85.9
	Medium	2.4	0.4	16.7	2.8	0.3	9.9	5.8	0.4	6.7	9.0	0.3	3.2	0.1	27.1
	High	2.1	0.3	15.0	3.5	0.2	5.6	5.6	0.2	3.8	6.3	0.1	2.3	0.2	53.8
Smoking pattern*	Never	10.9	2.4	22.0	12.7	2.7	20.9	11.6	1.0	8.9	14.9	0.4	2.8	2.0	82.6
	Former	4.9	1.1	21.6	6.2	1.1	18.2	7.8	1.3	16.1	5.5	0.2	3.8	0.9	80.4
	Current	2.4	1.1	44.8	1.6	0.5	32.6	2.6	0.7	25.9	2.1	0.2	9.8	0.9	80.4

*Taken from DMS waves except for 2030

## Discussion

Based on the present study, the prevalence of edentulism in Germany decreased by over 50% between 1997 and 2014, and will decrease once more by > 65% until 2030 compared with 2014. Being edentulous was associated with female sex, being a former or current smoker, and having a lower educational level. The largest impact on edentulism was found for the cohort effect (measured via the wave of the DMS). The reduction in edentulism prevalence was highly equitable, as both the relative and the absolute decrease were higher in groups with lower and medium versus high education. Vice versa, smokers (especially current ones) benefitted significantly less from the decline, as did females compared with males.

While our study only looked at a specific age group, the observed relative changes in the prevalence of edentulism and the number of prevalent cases can be compared with those reported from other surveys, some of which evaluated edentulism across the total population. The described decline in edentulism prevalence is in line with findings from a range of countries over the last 20–40 years; a 84% relative reduction was found in the UK (prevalence decreased from 37 to 6%) [17, 18], 57% in Finland (14–6%) [19], 61% in Australia (from 21 to 7%) [20], and 84% in Sweden (from 19 to 3%) [21, 22]. We predicted a further decline by > 65% in Germany between 2014 and 2030 (equaling 4.1% per year). This is in line with projections for the USA which predict a further decline by 44% between 2020 and 2030 (i.e., 4.4% per year) [6]. Notably, in the USA, the absolute decline was projected to be far lower (only 30% over the period), mainly as population growth will compensate these effects to some degree. This was not the case in our study (the absolute number of edentate individuals was expected to decrease by nearly 70% between 2014 and 2030, from 2.7 to 0.9 million). Of course, recent immigration waves might change these estimates.

While there seems to be general agreement as to the decline (and also its relative magnitude), it is not expected to be equally distributed between populations (as it has not been in the past). We found a number of socio-educational and demographic parameters to be associated with edentulism. Those with lower educational level, smokers, and females showed higher risk of edentulism and are projected to do so in the future. While it needs highlighting that, in contrast to other countries like the USA, those with lower education have benefitted the most from the decline [6], their risk of being edentulous will remain nearly 4 times as high compared with those with higher education in 2030 in Germany. There are a number of explanatory pathways for this inequality. Education has been found to impact on income, driving the financial means to keep their teeth healthy (attend privately paid prophylaxis programs and pay for expensive tooth-retaining therapies including, for example, root-canal or prosthetic treatment). Education also shapes behavior and decision-making; it is thus also possible that not only the dental self-care (associated with health literacy), but also the willingness to remove teeth might differ between educational levels.

Moreover, while the relative decline has been similar in the upper versus the lower arch, significant differences in the prevalence of edentulism between both arches emerged, with higher prevalences in the upper arch. This may have a number of reasons. First, both upper molars but also incisors have been found more prone for periodontal disease and associated tooth loss, a major source of tooth loss in Germany, than their lower arch counterparts [23]. Second, dental treatment decisions may have played a role, with rendering the upper arch edentulous being found less grave given the higher chances of providing satisfactory upper full dentures than doing the same so in the lower arch. It remains beyond this study to explore these underlying reasons, while it is relevant to note that the arch-specific edentulism has treatment consequences, too, and will probably continue into the future (something which was beyond this study to assess).

Across the globe, women have been found to be at higher risk for edentulism and tooth loss. It should be noted that this risk has been, globally, decreasing [5, 24]. Similarly, smoking has been unambiguously reported to increase the risk of tooth loss and edentulism [25, 26]. Similarly, periodontitis is an important intermediate factor in the association between smoking and tooth loss [27]. Reductions in edentulism in seniors in Germany within the last decade, however, were likely to happen after a re-organization of subsidization of dentures in statutory health insurance: new treatments were included in the list of covered treatments like cover dentures; thus, maintaining remaining teeth became attractive instead of indicating complete dentures.

This study has a number of limitations, some of which overlap with those described in the first part of this report [7]. First, we only assessed edentulism in an older population (65–74 years in our analysis, and 60–80 years in our projection); neither the measured age-group specific nor the predicted prevalence rates can be compared with those yielded for the total population in other studies. We decided to focus on this age group, as edentulism was virtually absent in other (younger) groups in the three DMS waves, which consequently would not have contributed to any statistical modeling but rather decreased the accuracy of our projections. Second, we could not assess edentulism in the very old, as only the last wave of the DMS had sampled those aged 75–100 years. Notably, the prevalence was remarkably higher (32.8%) in older seniors than in the younger senior age group in this wave [13, 14] and was significantly polarized: Those who received long-term care assistance (which are those who are not fully independent any longer) showed significantly increased risks of edentulism (53.7%). This is worrisome, as this group is projected to grow with time, but will be largely inaccessible for complex dental treatment (at least when in long-term care facilities) [28–30]. Future studies should attempt to reflect this group in both retrospective analyses and projections into the future. Third, we only evaluated edentulism as the final endpoint of all dental diseases. The preceding steps of sequential tooth loss had been evaluated in the first part of this report [7]. Fourth, our projection is only as good as the predictions it is based on; new developments will also change our estimates to a certain degree. This explicitly also includes demographic dynamics, introduced for example by the increased immigration into Germany in recent years. Given that the few data available on this group indicate a higher loss of teeth and a higher prevalence of edentulism compared with non-refugee individuals (notably with very limited sample sizes in the senior group aged 65–74 years), it could be speculated that this might to some degree dampen the extrapolated decrease [31]. Note, however, that refugee numbers have significantly decreased since 2015, and any assumptions built on these data will be highly prone to err. Similar, changes in the healthcare regulations, including the coverage and remuneration of tooth retaining and tooth replacing therapies, will impact on the future prevalence of edentulism by influencing both patients’ and dentists’ decision-making, which also determines the efforts placed into retaining teeth (or not). Last, it should be noted that the predictive value of the performed regression analysis (indicated as *R*^2^) was moderate at best, as discussed [7]. A large part of the variance in edentulism was explained by other non-identified factors. The model is thus only limitedly suitable to predict edentulism on individual level, while it seems suitable for the purpose of population-level predictions (as was done and validated here).

In conclusion and within the limitations of this study, edentulism has substantially declined between 1997 and 2014 in Germany, with those from lower educational level as well as those not smoking benefitting the most in relative terms. This decline is expected to continue until 2030. We projected the observed socio-educational and demographic inequalities of edentulism prevalence to continue until 2030. Given that edentulism has been found to negatively impact on systemic health of the aged, including a higher mortality and physical and mental impairment, our analyses are reassuring with regard to the generally and equitable improvements in the oral health of the German population. Nevertheless, further efforts are needed to tackle the underlying risk factors (mainly smoking), to facilitate tooth retention until high age, and to manage edentulism appropriately.
